# Septic Arthritis of the Shoulder Complicating Pregnancy

**DOI:** 10.1155/2014/738153

**Published:** 2014-05-18

**Authors:** Sara Raiser, Kathryn Davidson, Ashley Walsh, Robert Egerman

**Affiliations:** Department of Obstetrics and Gynecology, University of Florida, Gainesville, FL 32610, USA

## Abstract

Septic arthropathy leads to rapid joint destruction, impairment, and disability. *Staphylococcus* can be particularly virulent to bone and joints leading to adverse obstetric events. At 28 of weeks gestation, a patient presented with pyelonephritis and progressive left shoulder pain. Magnetic resonance imaging indicated early clavicular destruction and acromial involvement. Glenohumeral joint aspiration produced *Staphylococcus aureus*. The patient then had premature rupture of membranes and progressed rapidly to preterm delivery. Placental pathology revealed chorioamnionitis and microabscesses. Treatment of the infected joint required further surgical drainage and bone resection as well as extended antibiotics. It is important to remember that joint pain in pregnancy may indicate infective arthritis, and pyelonephritis can be a source of such an infection. Evaluation includes magnetic resonance imaging and consultation for joint aspiration. Prompt recognition and treatment are necessary to prevent joint destruction.

## 1. Introduction

Septic arthritis, an unusual complication of pregnancy, can quickly lead to irreversible arthropathy and disability. As musculoskeletal pain is common during pregnancy, the obstetrical provider needs to be vigilant regarding historical and clinical findings suggesting infection. We describe septic arthritis of the shoulder in the setting of pyelonephritis in pregnancy.

## 2. Case

A 30-year-old white female at 28 weeks of gestation presented to an outside emergency room with flank pain, nausea, and fever. Her past medical history included a previous term delivery and a surgically treated ectopic pregnancy. She worked as a phlebotomist and denied a needle stick injury or intravenous illicit drug use. Her prenatal laboratory assessment was negative for sexually transmitted infections as well as asymptomatic bacteriuria. Her personal and family medical histories were negative. Urinalysis at that time showed moderate bacteria, negative nitrites and leukocyte esterase, 1+ blood, and specific gravity of 1.025. A renal ultrasound demonstrated no nephrolithiasis or hydronephrosis and the patient was treated with intravenous hydration, narcotic pain control, and discharged home.

Complaining of persistent back pain and fever, the following day, she was admitted to a local hospital. The urine culture sent previously resulted in 10–100,000 colonies/mL of mixed urogenital flora; thus, she was diagnosed with and treated for pyelonephritis with cefazolin. She started to report some left shoulder pain that worsened over the next day and localized to the superior anterior shoulder in the vicinity of the acromioclavicular (AC) joint. Left glenohumeral joint aspiration produced 1.5 mL of purulent fluid with staphylococci being present, prompting the outside hospital to change her antibiotic from cefazolin to vancomycin. Physical exam at that time demonstrated an obese female (BMI 38 kg/m^2^) with temperature of 39.6°C, pulse 144 beats per minute, and blood pressure 185/72 mmHg (isolated). Laboratory analysis showed a mild leukocytosis of 13,000/mm^3^ with 38% band neutrophils, hemoglobin 9.8 g/dL, hematocrit 29.3%, platelets 69,000/mm^3^, and creatinine 0.68 mg/dL. The following day, she had preterm premature rupture of membranes and preterm labor prompting transfer to our hospital 4 days after her initial presentation. Soon after arrival, she spontaneously delivered a vigorous female infant.

Vital signs on arrival included a temperature of 36.9, blood pressure of 131/72, and heart rate of 74. Examination of the chest, heart, and abdomen was benign aside from right flank pain. The oropharynx was clear, and there was no tooth decay or gingivitis. Skin examination indicated intravenous cannulas in both arms without inflammation. The musculoskeletal assessment demonstrated diffuse swelling along the left shoulder and AC joint with no erythema. There was marked tenderness and decreased range of motion due to pain, although sensation and muscle strength remained intact. Elevation of the arm forward to 90 degrees with adduction across the upper chest elicited a positive cross arm flexion test.

**Figure 1:**
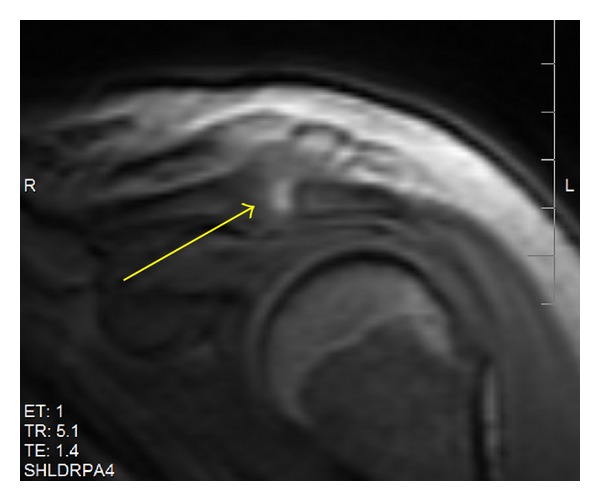
Left shoulder magnetic resonance imaging revealing edema within the acromioclavicular (AC) joint and edema and fluid outside of the joint. There is distal clavicular cortical destruction (arrow).

Laboratory analysis on the day of transfer included a normal coagulation profile and a negative urine drug screen. The white blood cell count was 10,900/mm^3^, 82% neutrophils, and no bands were noted. Hematocrit was stable at 29% and platelets were 111,000/mm^3^. Blood cultures were sent and quickly were positive for methicillin sensitive* Staphylococcus aureus* that matched the organism recovered from the previously performed joint aspirate based on staphylococcal type and sensitivities. Radiography of the chest as well as transthoracic and transesophageal echocardiography was normal. Noncontrast magnetic resonance imaging of the left shoulder showed fluid within the AC joint and edema and fluid outside of the joint with no abscess formation. There was cortex destruction of the distal clavicle with possible acromial involvement. The glenohumeral joint was not involved and the impression was septic arthritis of the AC joint (Figure 1).

Placental pathology showed likely placental abruption with intervillous and subchorionic thrombi. Additionally, there was fetal surface vasculitis, acute chorioamnionitis, and intervillous abscesses. These microabscesses appeared most consistent with hematogenous spread of the intravascular bacteria. The neonatal cultures did not grow organisms, although antibiotic coverage was provided.

Postpartum, despite directed antibiotic therapy, the patient still complained of shoulder pain and had persistent leukocytosis. On postpartum day five, incision and drainage of the glenohumeral joint and resection of the distal clavicle were performed for septic arthritis and osteomyelitis. Blood and joint cultures obtained at this time later resulted as no growth. Three days after this procedure, her white blood cell count normalized and her pain improved and she was discharged on intravenous antibiotics for additional six weeks.

## 3. Comment

Septic arthropathy is an unusual complication of pregnancy, particularly when the shoulder, acromion, or clavicle is involved. The causative organism is typically* Staphylococcus aureus* [1]. Predisposing factors to septic arthritis pertinent to the gravida include diabetes, rheumatoid arthritis, intravenous drug abuse, a prior intra-articular steroid injection, or trauma. Most cases of infectious arthritis in pregnancy involve the sacroiliac joints [2]. We have found only one other case report of an infected shoulder joint complicating pregnancy and presenting as an orthopedic emergency [3]. The authors reported on an infected shoulder joint in a patient who presented four days after elective termination with fever and shoulder pain. Arthrocentesis produced purulent fluid and treatment required arthroscopic irrigation and debridement.* Streptococcus agalactiae* was the causative organism.

Several prior reports associate urinary tract infection with septic arthropathy. The mechanism includes a microtrauma of the joint seeded by concomitant bacteremia which may or may not be clinically noticed. The urine culture in this case revealed only mixed flora, disparate from the blood and joint cultures. Because of the concurrent timing of the patient's clinical symptoms of flank and joint pain, the urinary tract may in fact have been the source despite the uninformative urinary culture. Another less likely potential source of infection in this case would be from the skin via a catheter-associated bacteremia.

Joint pain in pregnancy covers a wide differential diagnosis including trauma, bacterial or viral (parvovirus and hepatitis b or c) arthropathy, collagen vascular disease (systemic lupus erythematosus or rheumatoid arthritis), or enteropathic (inflammatory bowel disease) arthritis. Smaller and more distal joints are easier to assess than larger more central joints. Nonetheless, joint destruction can occur with either. The patient's history and concomitant illness provide useful clues as to the origins of the arthritis.

Magnetic resonance imaging is suited for a detailed assessment of the inflammatory joint [4]. Joint aspiration should be performed in all cases of septic arthritis, as this can help tailor treatment and allow drainage of large collections to facilitate recovery. Initial therapy begins with broad antibiotic coverage to include gram-positive and gram-negative organisms which can then be adjusted based on culture sensitivities [1]. Recommendations for duration of therapy vary but typically involve many weeks.

For the obstetric provider, we encourage cognizance of infectious arthropathy and the need for early consultation and imaging in patients with joint pain and UTI in pregnancy in order to prevent adverse outcomes [5, 6].
